# Response to Olgun et al.: Bronchoscopy During Pediatric Extracorporeal Membrane Oxygenation

**DOI:** 10.1177/08850666261469113

**Published:** 2026-07-13

**Authors:** Melany Gaetani, Briseida Mema

**Affiliations:** 1 Interdepartmental Division of Critical Care, 7979 University of Toronto, Toronto, Canada; 2 Department of Critical Care Medicine, The Hospital for Sick Children, Toronto, Canada; 3 Department of Paediatrics, University of Toronto, Toronto, Canada

We thank Olgun and colleagues for their thoughtful comments regarding our recent study of bronchoscopy in critically ill children. We appreciate their interest in the Extracorporeal Membrane Oxygenation (ECMO) subgroup within our cohort and their recognition of the limited literature describing bronchoscopy practices and outcomes in children receiving ECMO support. Their comments provide an opportunity to further characterize our ECMO cohort and address several important questions regarding procedure-related complications, the influence of bronchoscopy on clinical management, the potential impact on ECMO support and decannulation, and practical considerations surrounding bronchoscopy in anticoagulated patients receiving ECMO support.

## ECMO-specific complications

In our cohort, 35 patients underwent 63 bronchoscopic procedures while receiving ECMO. Complications were documented following 13 procedures (20.6%), the majority of which were minor and self-limited.

The most common adverse event was airway bleeding, occurring in 6 procedures (9.5%) and generally in the setting of friable mucosa, pulmonary hemorrhage, or hemoptysis. Transient oxygen desaturation occurred in 3 procedures (4.8%) and resolved with temporary interruption of the procedure and supportive measures. One patient (1.6%) experienced transient hypotension responsive to fluid administration, while another (1.6%) developed transient hypertension following endobronchial vasopressor administration during treatment of active airway hemorrhage. Airway trauma or suction-related injury was observed in 1 procedure (1.6%). Two procedures (3.2%) were terminated early because of active bleeding, worsening oxygenation, or technical limitations related to airway obstruction. Importantly, no pneumothorax, clinically significant bradycardia, cannula complications, cardiac arrest, or procedure-related deaths occurred. Overall, these findings suggest that flexible bronchoscopy can be safely performed in carefully selected pediatric patients receiving ECMO support when undertaken by experienced multidisciplinary teams.

## Impact of bronchoscopy on clinical management

Bronchoscopy provided both diagnostic and therapeutic benefit in our ECMO cohort. Increased airway secretions were identified in 35 procedures (55.6%), while abnormal airway or mucosal findings, including inflammation, edema, friability, airway collapse, or abnormal anatomy, were observed in 30 procedures (47.6%). Airway obstruction due to mucus plugs, blood clots, casts, airway compression, or foreign bodies was identified in 17 procedures (27.0%).

Therapeutic interventions were performed in 19 procedures (30.2%), including airway clearance with mucus plug or clot removal and administration of endobronchial mucolytic therapy with DNase or pulmozyme in 8 procedures (12.7%). BAL specimens were obtained in 17 procedures (27.0%) and yielded microbiological organisms in several cases, most commonly Klebsiella spp. (6.3%), Candida spp. (4.8%), Streptococcus pyogenes (3.2%), and Stenotrophomonas maltophilia (3.2%). Bronchoscopy therefore contributed to both diagnostic evaluation and therapeutic decision-making in this complex patient population.

## Impact on ECMO Support and Decannulation

The median ECMO duration was 156 hours (6.5 days; IQR 120–228 hours [5.0–9.5 days]). Among bronchoscopies performed during ECMO, the median interval from bronchoscopy to decannulation was 7.5 days (IQR 3.0–33.3 days). Overall, 5 procedures (7.9%) occurred within 24 hours of decannulation, 11 (17.5%) within 48 hours, 14 (22.2%) within 72 hours, and 26 (41.3%) within 7 days, demonstrating that bronchoscopy was frequently performed throughout the ECMO course, including immediately preceding decannulation. 12 patients (34.3%) underwent multiple bronchoscopies while on ECMO, with one patient undergoing as many as eight procedures. This finding highlights that bronchoscopy was commonly used as a serial therapeutic modality for ongoing airway management during ECMO rather than as a single intervention.

Only one bronchoscopy (1.6%) was documented as performed to assess whether airway clearance could optimize the patient for ECMO decannulation. Nevertheless, bronchoscopy frequently identified potentially reversible airway pathology and enabled therapeutic interventions such as airway clearance and endobronchial mucolytic therapy, which may have contributed to respiratory optimization during ECMO. This retrospective study was not designed to determine whether bronchoscopy directly shortened ECMO duration or expedited decannulation.

## Practical Considerations for Bronchoscopy During ECMO

At our institution, all bronchoscopies performed during ECMO are undertaken at the bedside in the pediatric ICU by pediatric critical care attending physicians or fellows, with support from a multidisciplinary team including a respiratory therapist, ECMO specialist, and bedside nurse. The cardiovascular surgery team is routinely notified before the procedure and frequently attends when clinically indicated. This collaborative approach minimizes the risks associated with transport while facilitating safe procedural planning. In cases of active airway bleeding, topical vasopressor therapy was used for hemostasis when indicated. Although two procedures (3.2%) were terminated early because of bleeding, worsening oxygenation, or technical limitations, no pneumothorax, ECMO cannula complications, cardiac arrest, or procedure-related deaths occurred, supporting the safety of bronchoscopy when performed by an experienced multidisciplinary team.

## Conclusion

We thank Olgun and colleagues for highlighting the need for additional data regarding bronchoscopy during pediatric ECMO. We agree that careful patient selection and multidisciplinary expertise are essential and that bronchoscopy should not be avoided solely because a patient is receiving ECMO support. In our experience, bronchoscopy was associated with a low rate of major complications while providing important diagnostic information and facilitating therapeutic interventions that may improve airway patency and respiratory optimization. Although our study was not designed to determine its effect on ECMO duration or decannulation, our findings support the judicious use of bronchoscopy in appropriately selected patients. Prospective multicentre studies are needed to better define the optimal indications, timing, and impact of bronchoscopy on clinically important ECMO outcomes, including liberation from ECMO and mechanical ventilation.

